# Portable Differential Detection of CTX-M ESBL Gene Variants, *bla*_CTX-M-1_ and *bla*_CTX-M-15_, from Escherichia coli Isolates and Animal Fecal Samples Using Loop-Primer Endonuclease Cleavage Loop-Mediated Isothermal Amplification

**DOI:** 10.1128/spectrum.03316-22

**Published:** 2022-12-13

**Authors:** Owen Higgins, Alexandra Chueiri, Louise O'Connor, Sinéad Lahiff, Liam Burke, Dearbhaile Morris, Nicola Maria Pfeifer, Belén González Santamarina, Christian Berens, Christian Menge, Manuela Caniça, Vera Manageiro, Veljo Kisand, Marwa M. Hassan, Brian Gardner, Arnoud H. M. van Vliet, Roberto M. La Ragione, Bruno Gonzalez-Zorn, Terry J. Smith

**Affiliations:** a Molecular Diagnostics Research Group, School of Biological and Chemical Sciences, University of Galway, Galway, Ireland; b Antimicrobial Resistance and Microbial Ecology Group, School of Medicine, University of Galway, Galway, Ireland; c Centre for One Health, Ryan Institute, University of Galway, Galway, Ireland; d Friedrich-Loeffler-Institut, Federal Research Institute for Animal Health, Institute of Molecular Pathogenesis, Jena, Germany; e National Reference Laboratory of Antibiotic Resistances and Healthcare Associated Infections, Department of Infectious Diseases, National Institute of Health Dr. Ricardo Jorge, Lisbon, Portugal; f Institute of Technology, University of Tartu, Tartu, Estonia; g Department of Comparative Biomedical Sciences, School of Veterinary Medicine, Faculty of Health and Medical Sciences, University of Surrey, Guildford, Surrey, United Kingdom; h Department of Microbial Sciences, School of Biosciences and Medicine, Faculty of Health and Medical Sciences, University of Surrey, Guildford, Surrey, United Kingdom; i Antimicrobial Resistance Unit, Veterinary School and VISAVET, Complutense University of Madrid, Spain; Pontificia Universidade Catolica do Paraná

**Keywords:** AMR, CTX-M, ESBL, Enterobacteriaceae, LAMP

## Abstract

Cefotaximase-Munich (CTX-M) extended-spectrum beta-lactamase (ESBL) enzymes produced by *Enterobacteriaceae* confer resistance to clinically relevant third-generation cephalosporins. CTX-M group 1 variants, CTX-M-1 and CTX-M-15, are the leading ESBL-producing *Enterobacteriaceae* associated with animal and human infection, respectively, and are an increasing antimicrobial resistance (AMR) global health concern. The *bla*_CTX-M-1_ and *bla*_CTX-M-15_ genes encoding these variants have an approximate nucleotide sequence similarity of 98.7%, making effective differential diagnostic monitoring difficult. Loop-primer endonuclease cleavage loop-mediated isothermal amplification (LEC-LAMP) enables rapid real-time multiplex pathogen detection with single-base specificity and portable on-site testing. We have developed an internally controlled multiplex CTX-M-1/15 LEC-LAMP assay for the differential detection of *bla*_CTX-M-1_ and *bla*_CTX-M-15_. Assay analytical specificity was established using a panel of human, animal, and environmental Escherichia coli isolates positive for *bla*_CTX-M-1_ (*n* = 18), *bla*_CTX-M-15_ (*n* = 35), and other closely related *bla*_CTX-Ms_ (*n* = 38) from Ireland, Germany, and Portugal, with analytical sensitivity determined using probit regression analysis. Animal fecal sample testing using the CTX-M-1/15 LEC-LAMP assay in combination with a rapid DNA extraction protocol was carried out on porcine fecal samples previously confirmed to be PCR-positive for E. coli *bla*_CTX-M_. Portable instrumentation was used to further analyze each fecal sample and demonstrate the on-site testing capabilities of the LEC-LAMP assay with the rapid DNA extraction protocol. The CTX-M-1/15 LEC-LAMP assay demonstrated complete analytical specificity for the differential detection of both variants with sensitive low-level detection of 8.5 and 9.8 copies per reaction for *bla*_CTX-M-1_ and *bla*_CTX-M-15_, respectively, and E. coli *bla*_CTX-M-1_ was identified in all *bla*_CTX-M_ positive porcine fecal samples tested.

**IMPORTANCE** CTX-M ESBL-producing E. coli is an increasing AMR public health issue with the transmission between animals and humans via zoonotic pathogens now a major area of interest. Accurate and timely identification of ESBL-expressing E. coli CTX-M variants is essential for disease monitoring, targeted antibiotic treatment and infection control. This study details the first report of portable diagnostics technology for the rapid differential detection of CTX-M AMR markers *bla*_CTX-M-1_ and *bla*_CTX-M-15_, facilitating improved identification and surveillance of these closely related variants. Further application of this portable internally controlled multiplex CTX-M-1/15 LEC-LAMP assay will provide new information on the transmission and prevalence of these CTX-M ESBL alleles. Furthermore, this transferable diagnostic technology can be applied to other new and emerging relevant AMR markers of interest providing more efficient and specific portable pathogen detection for improved epidemiological surveillance.

## INTRODUCTION

Beta-lactamases are bacterial hydrolytic enzymes that cleave the amide bond of four-membered lactam rings providing resistance to beta-lactam antibiotics, the most widely used class of antimicrobials (1, 2). Classification of beta-lactamase enzymes is based on molecular size and active-site amino acid homology resulting in four Ambler Classification groups that can be divided into three serine-beta-lactamase groups (A, C, D) and one metallo-beta-lactamase group (B) (3). Extended-spectrum beta-lactamase (ESBL) enzymes are the most abundant beta-lactamases belonging primarily to the Ambler Class A serine-beta-lactamase group. ESBLs confer resistance to third-generation cephalosporins, such as cefotaxime, ceftriaxone, cefixime, and ceftazidime, antimicrobials considered critically important to both human and animal health (4, 5). During the 1980s, there was a rapid increase in the emergence of ESBLs with SHV and TEM types predominantly reported. However, since the first report of clinical cefotaxime-resistant E. coli strains from non-TEM or non-SHV ESBLs in Germany in 1989 (6), Cefotaximase-Munich (CTX-M) has become the dominant global ESBL in *Enterobacteriaceae*, associated with human and animal infection (7).

CTX-M enzymes originated from the mobilization of *Kluyvera* spp. chromosomal beta-lactamase genes integrating with mobile plasmids, with later CTX-M variants emerging from point mutations associated with antibiotic selective pressure (6). These enzymes are natural cephalosporinases that have increased activity against cefotaxime and ceftriaxone antibiotics with additional resistance to other extended-spectrum cephalosporins such as cefepime and ceftazidime (8). Typically, CTX-M genes are located on transferable conjugative plasmids flanked by insertion sequences promoting mobility and strong expression; however, reports of chromosomal location are becoming more frequent (9–11). Based on amino acid homology, CTX-Ms are classified into five phylogenetic families (1, 2, 8, 9, and 25) and named after the first member of each group (12), with over 230 types identified to date.

CTX-Ms are the predominant ESBL enzymes in *Enterobacteriaceae* with a principal host being E. coli, a commensal opportunistic zoonotic pathogen common to the intestinal microbiota of humans and animals (13). Primary reservoirs for CTX-M-producing E. coli are hospitals, food-producing animals (porcine, bovine, poultry) and derived products, companion animals, and associated environments (14, 15). E. coli beta-lactamase (*bla*) CTX-M genes most commonly associated with animal and human infection, respectively, are CTX-M group 1 gene variants, *bla*_CTX-M-1_ and *bla*_CTX-M-15_ (16, 17). Although these variants have an approximate nucleotide sequence similarity of 98.7%, they can often produce varying hydrolytic activity toward certain cephalosporin and fluoroquinolone antibiotics (18–20). The comprehensive dissemination of *bla*_CTX-M-15_ among humans is directly linked to its association with E. coli ST131-O25b, a member of the highly virulent B2 phylogenetic group, responsible for over half of all ESBL E. coli isolates. E. coli ST131-O25b incorporates multidrug-resistant plasmids containing multiple antimicrobial resistance (AMR) genes that typically cause serious infections with limited treatment options to which carbapenems are often used as a last resort resulting in increasing cases of carbapenem resistance (21).

Application and misuse of broad-spectrum antimicrobials is a leading contributor to AMR selection and dissemination (5), and the increasing prevalence of CTX-M-1 and CTX-M-15-producing E. coli is now a global health concern with transmission between animals and humans via zoonotic pathogens a major area of interest (13). The World Health Organization (WHO) has recently identified cephalosporin-resistant *Enterobacteriaceae* as priority pathogens of critical importance (22), with the WHO Global Action Plan aiming to address the threat of AMR using a One Health approach (23). The One Health concept involves collaboration of multidisciplinary health science sectors on local and international levels to achieve optimal health for people, animals, and the environment. A key element to the One Health approach for AMR is the surveillance of relevant markers to help monitor current resistance levels and associated health burdens, as well as identifying emerging trends of clinical significance (5).

Accurate identification of ESBL-producing E. coli CTX-M-1 and CTX-M-15 variants is essential to aid targeted antibiotic treatment and disease monitoring, with timely detection enabling improved AMR surveillance and infection control. Phenotypic culture-based diagnostics for the identification of CTX-M-producing E. coli is traditionally performed using media dilution or disc diffusion assays to determine antibiotic susceptibility or resistance profiles (24). These methods often require 24 h to 48 h and typically only differentiate ESBLs from non-ESBL producers without identifying actual CTX-M types due to varied enzyme expression or limited antibiotic specificity (24). Genotypic molecular-based diagnostics such as PCR and DNA sequencing offer more rapid and specific identification of CTX-M genes and gene variants (25). However, due to limitations such as expense, instrumentation, and complexity, these methods are often outsourced or not suitable for portable on-site application. Furthermore, the nucleotide sequence similarity between *bla*_CTX-M-1_ and *bla*_CTX-M-15_ makes accurate differential detection with conventional molecular diagnostics difficult.

**FIG 1:**
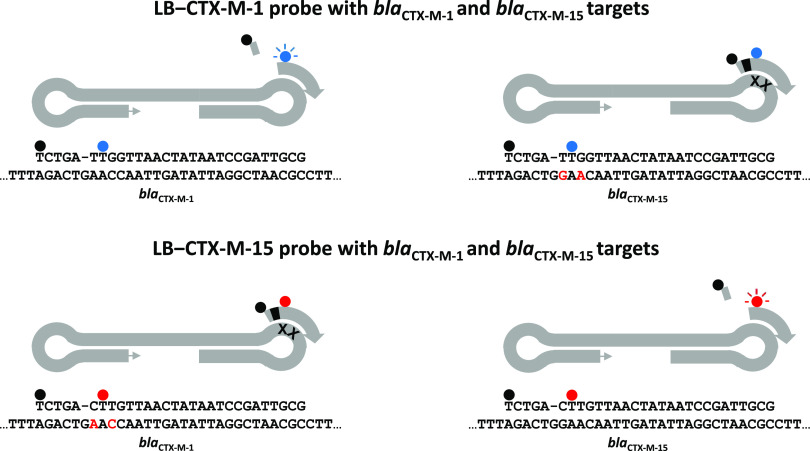
Mechanism of CTX-M-1/15 LEC-LAMP assay target detection and differentiation. The fluorophore (blue/red) and quencher (black) labeled LEC-LAMP probes hybridize to complementary loop targets in the LAMP amplicon. Complete probe to target complementarity, as per LB–CTX-M-1 with *bla*_CTX-M-1_ or LB–CTX-M-15 with *bla*_CTX-M-15_, is required for probe abasic site cleavage and subsequent dye-label dissociation fluorescence production. Nucleotide mismatches adjacent to the probe abasic site, as per LB–CTX-M-1 with *bla*_CTX-M-15_ or LB–CTX-M-15 with *bla*_CTX-M-1_, inhibits cleavage and prevents dye-label dissociation fluorescence production, enabling target differentiation.

**FIG 2:**
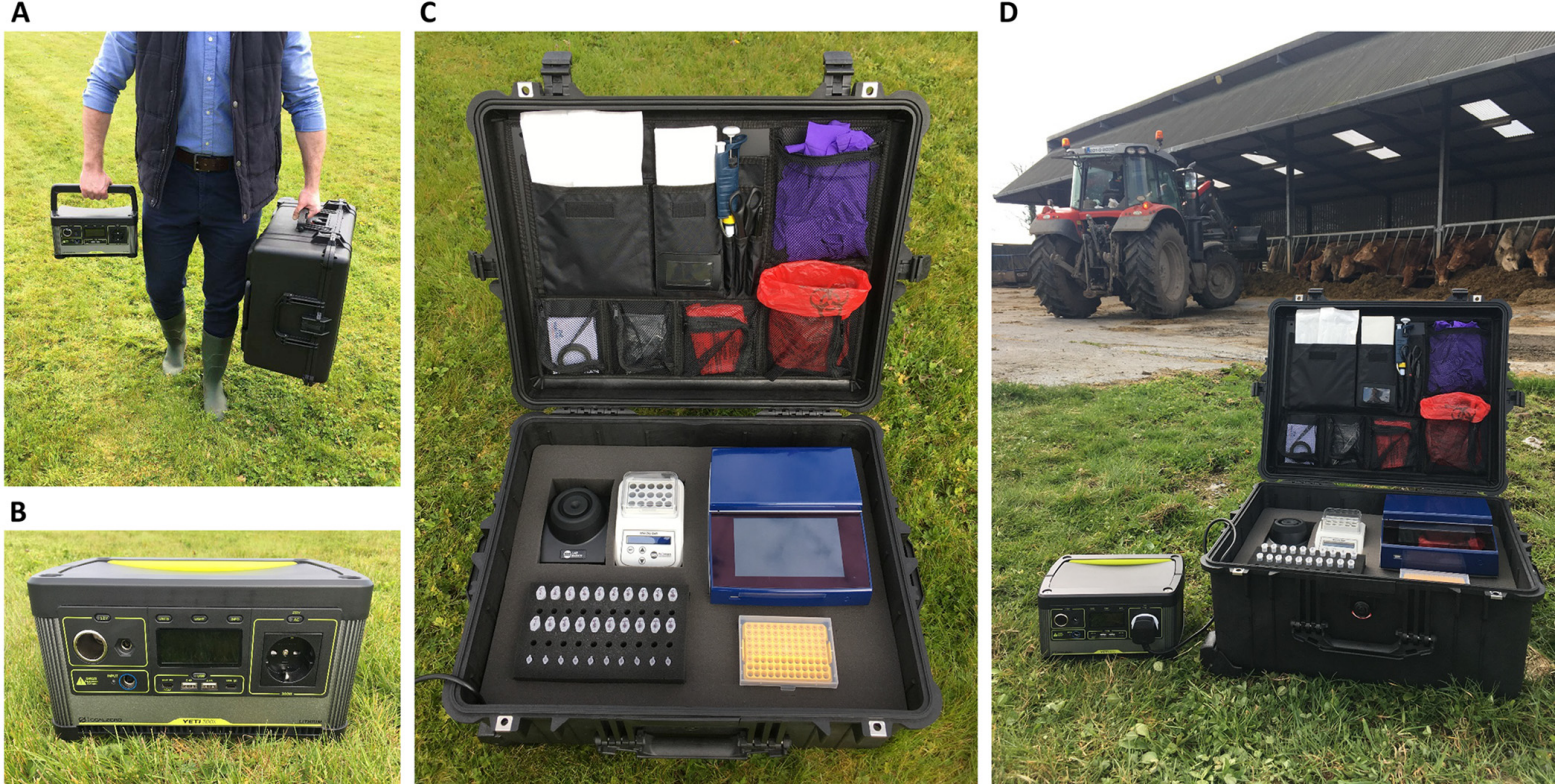
Portable diagnostics workstation. (A) Manual workstation transport. (B) Goal Zero power source. (C) Mobile workstation containing thermostatic fluorometer, mini dry bath, vortex, protector case, laboratory accessories and reagents for DNA extractions and LEC-LAMP reactions. (D) On-site application.

**FIG 3:**
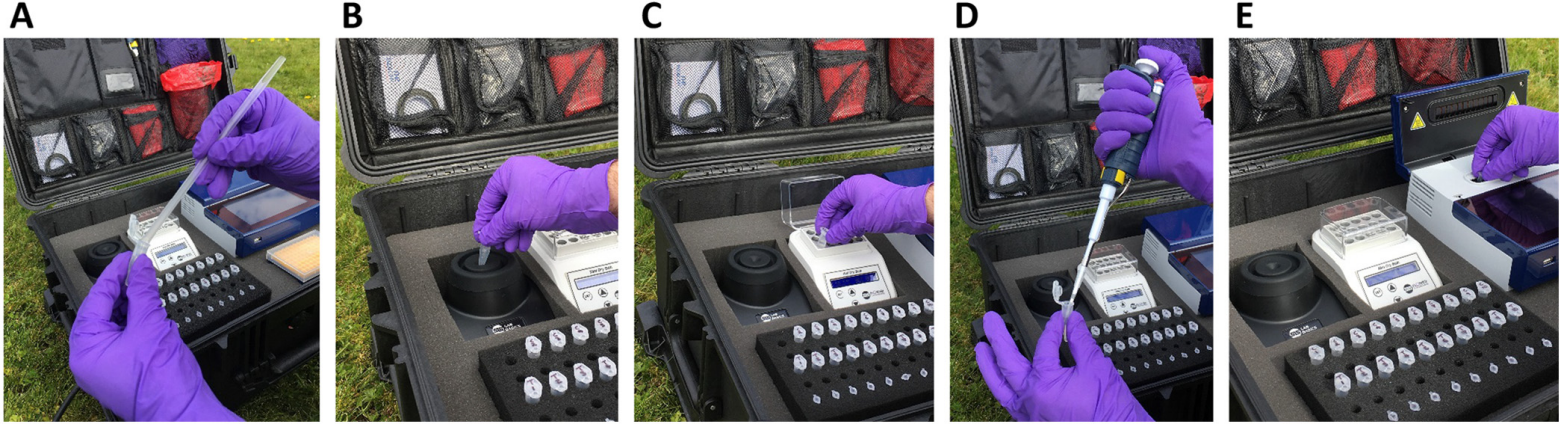
On-site fecal sample analysis workflow. (A) Fecal sample transfer to preheated extraction reagent using sterile spatula. (B) Sample suspension vortex. (C) Sample heat lysis. (D) Lysate transfer to neutralization buffer. (E) Transfer of extracted DNA to LEC-LAMP reactions followed by analysis.

Loop-primer endonuclease cleavage loop-mediated isothermal amplification (LEC-LAMP) is a recently developed diagnostic technology that enables multiplex pathogen detection and single nucleotide polymorphism (SNP) identification with portable on-site testing applications (26). In this study, an internally controlled multiplex CTX-M-1/15 LEC-LAMP assay (Table 1) for the differential detection of AMR markers *bla*_CTX-M-1_ and *bla*_CTX-M-15_ in a single reaction (Fig. 1) was developed and validated. This technology was evaluated in combination with a rapid DNA extraction protocol using porcine fecal samples previously confirmed to be PCR-positive for E. coli *bla*_CTX-M_. Proof-of-concept demonstration for the portable on-site application of this technology was performed using a custom mobile diagnostics workstation (Fig. 2 and 3).

**TABLE 1:** LEC-LAMP Oligonucleotides^*a*^

Type	Sequence (5′ to 3′)
CTX-M-1/15 LEC-LAMP assay	
FIP	GCTTTCACTTTTCTTCAGCACCGC ~ CGCTTTGCGATGTGCAG
BIP	CCGAATCTGTTAAATCAGCGAGTTGAGA ~ GACGTGCTTTTCCGC
F3	GTGGCATTGATTAACACAGCAG
B3	GCGCTAAGCTCAGCCAG
LF	CGGCCATCACTTTACTGGTGC
LB–CTX-M-1	(BHQ1)TCTGA(dSpacer) **T** (FAMdT) **G**GTTAACTATAATCCGATTGCG
LB–CTX-M-15	(BHQ1)TCTGA(dSpacer) **C** (HEXdT) **T**GTTAACTATAATCCGATTGCG
IAC LEC-LAMP assay	
FIP	ATATCCGCGATCCTTGCGTTGT ~ TCCCCGCTATGGAAGGTC
BIP	CACCTGTTCGTGTCGTATCGGT ~ ATGCATTACCAGAGTGCTCC
F3	TACAGCGAAAAGCCCAGC
B3	AAGCGACGAATGTCCTGTG
LF	TCTTAATTGCTTGCCGGAGC
LB–IAC	(BHQ2)ACTC(dSpacer)CA(Cy5dT) GCGCAATGATGGTATAATCC

a ~, separation between sense and antisense FIP and BIP sequences; BHQ1, black hole quencher 1; dSpacer, 1’ 2’-dideoxyribose; bold and underline, nucleotide differences between LB–CTX-M-1 and LB–CTX-M-15; FAMdT, thymine-linked 6-carboxyfluorescein fluorophore; HEXdT, thymine-linked 6-hexachlorofluorescein fluorophore; BHQ2, black hole quencher 2; Cy5dT, thymine-linked cyanine fluorophore.

## RESULTS

### CTX-M-1/15 LEC-LAMP assay target detection and differentiation.

**FIG 4:**
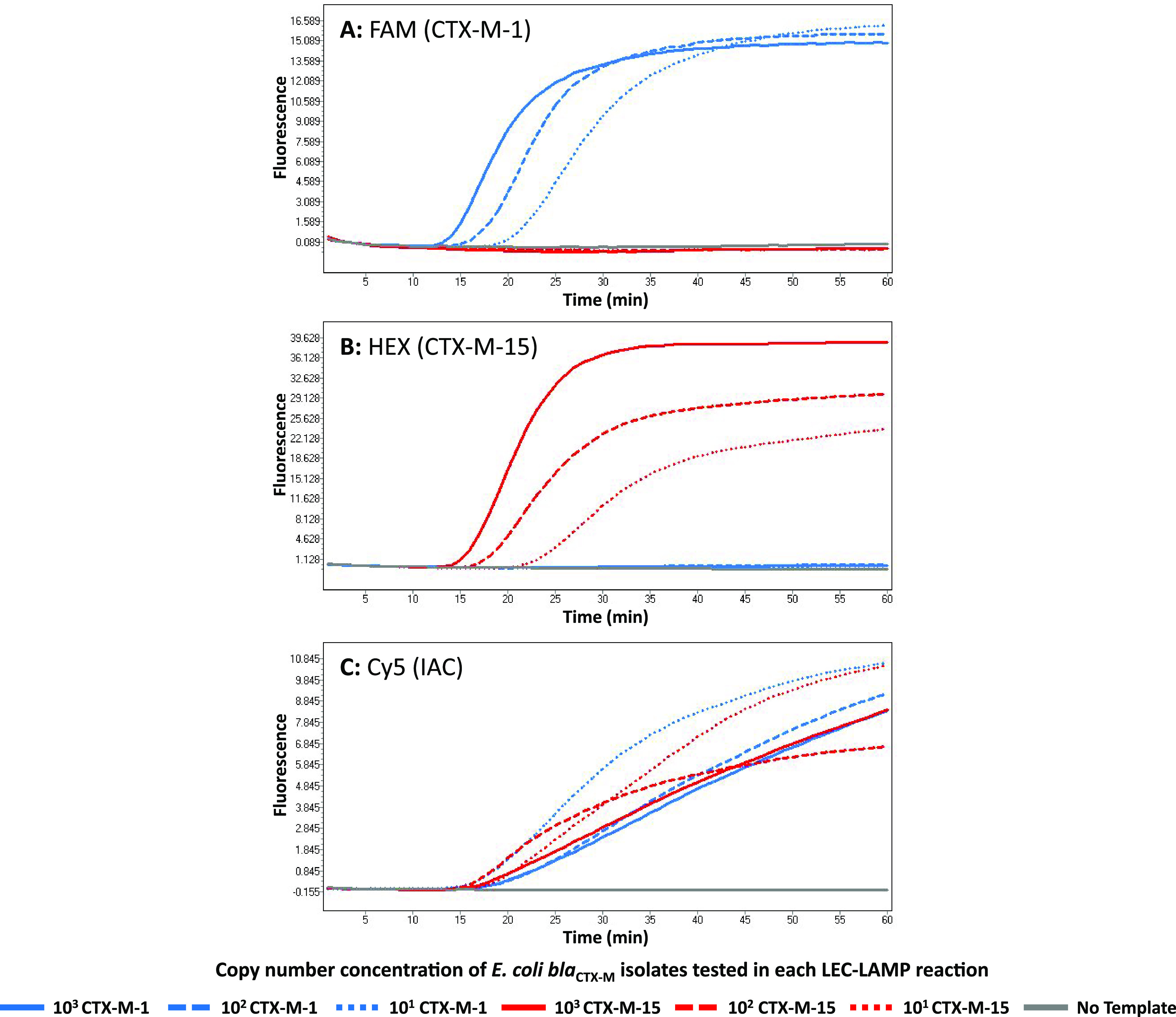
CTX-M-1/15 LEC-LAMP assay target detection and differentiation. The internally controlled CTX-M-1/15 LEC-LAMP assay was tested with purified DNA from E. coli isolates positive for *bla*_CTX-M-1_ or *bla*_CTX-M-15_ at 10^3^, 10^2^, and 10^1^ copies, in the presence of 500 copies IAC DNA template. Reactions were performed at 63°C in a LightCycler 480 using fluorescence detection channels: FAM, CTX-M-1 specific (A); HEX, CTX-M-15 specific (B); and Cy5, IAC specific (C). Resulting amplification curves indicate sensitive differential detection of *bla*_CTX-M-1_ and *bla*_CTX-M-15_ with simultaneous IAC detection in each reaction.

The internally controlled CTX-M-1/15 LEC-LAMP assay successfully demonstrated specific differential target detection of the CTX-M group 1 gene variants, *bla*_CTX-M-1_ and *bla*_CTX-M-15_ (Fig. 4). E. coli *bla*_CTX-M-1_ purified genomic DNA tested at 10^3^, 10^2^, and 10^1^ copies per reaction was detected in the CTX-M-1 specific FAM detection channel (Fig. 4A, blue), with no detection for this target observed in the CTX-M-15 specific HEX detection channel (Fig. 4B, blue). Conversely, E. coli *bla*_CTX-M-15_ purified genomic DNA tested at 10^3^, 10^2^, and 10^1^ copies per reaction was correctly detected in the corresponding HEX detection channel (Fig. 4B, red), with no detection for this target observed in the FAM detection channel (Fig. 4A, red). Successful co-amplification and detection of the IAC DNA template tested at 500 copies in each bacterial target reaction can be observed in the appropriate Cy5 detection channel (Fig. 4C). Bacterial and synthetic IAC target time-to-detection values ranged from 10 to 20 min. The no template control reaction performed successfully as no corresponding signal was observed in any detection channel (Fig. 4A to C, gray).

### CTX-M-1/15 LEC-LAMP assay analytical specificity and sensitivity.

Complete analytical specificity for the differential detection of CTX-M group 1 gene variants, *bla*_CTX-M-1_ and *bla*_CTX-M-15_, was demonstrated using the CTX-M-1/15 LEC-LAMP assay (Table S3). All inclusivity panel strains of E. coli *bla*_CTX-M-1_ positive isolates (*n* = 18) and E. coli *bla*_CTX-M-15_ positive isolates (*n* = 35) were correctly detected in corresponding FAM and HEX detection channels, respectively, with no nonspecific detection observed. All exclusivity panel strains of other closely related E. coli *bla*_CTX-M_ isolates (*n* = 38) did not produce fluorescence signal in any detection channels, with the exception of E. coli *bla*_CTX-M-55_ which was detected in the HEX (CTX-M-15 specific) detection channel. In the event of an E. coli isolate not being detected in either the FAM or HEX detection channels, the IAC DNA template was successfully detected in the Cy5 (IAC specific) detection channel validating each reaction.

Analytical sensitivity testing for the internally controlled CTX-M-1/15 LEC-LAMP assay established an LOD for each CTX-M group 1 gene variant, *bla*_CTX-M-1_ and *bla*_CTX-M-15_. Table S4 highlights the E. coli *bla*_CTX-M-1_ and E. coli *bla*_CTX-M-15_ copy number concentrations and replicates tested, indicating the number replicates detected or not detected. Resulting data were used in combination with Minitab 17 to perform probit regression analysis establishing an assay LOD with 95% confidence of 8.5 and 9.8 copies per reaction for *bla*_CTX-M-1_ and *bla*_CTX-M-15_, respectively.

### Laboratory animal fecal sample testing.

**FIG 5:**
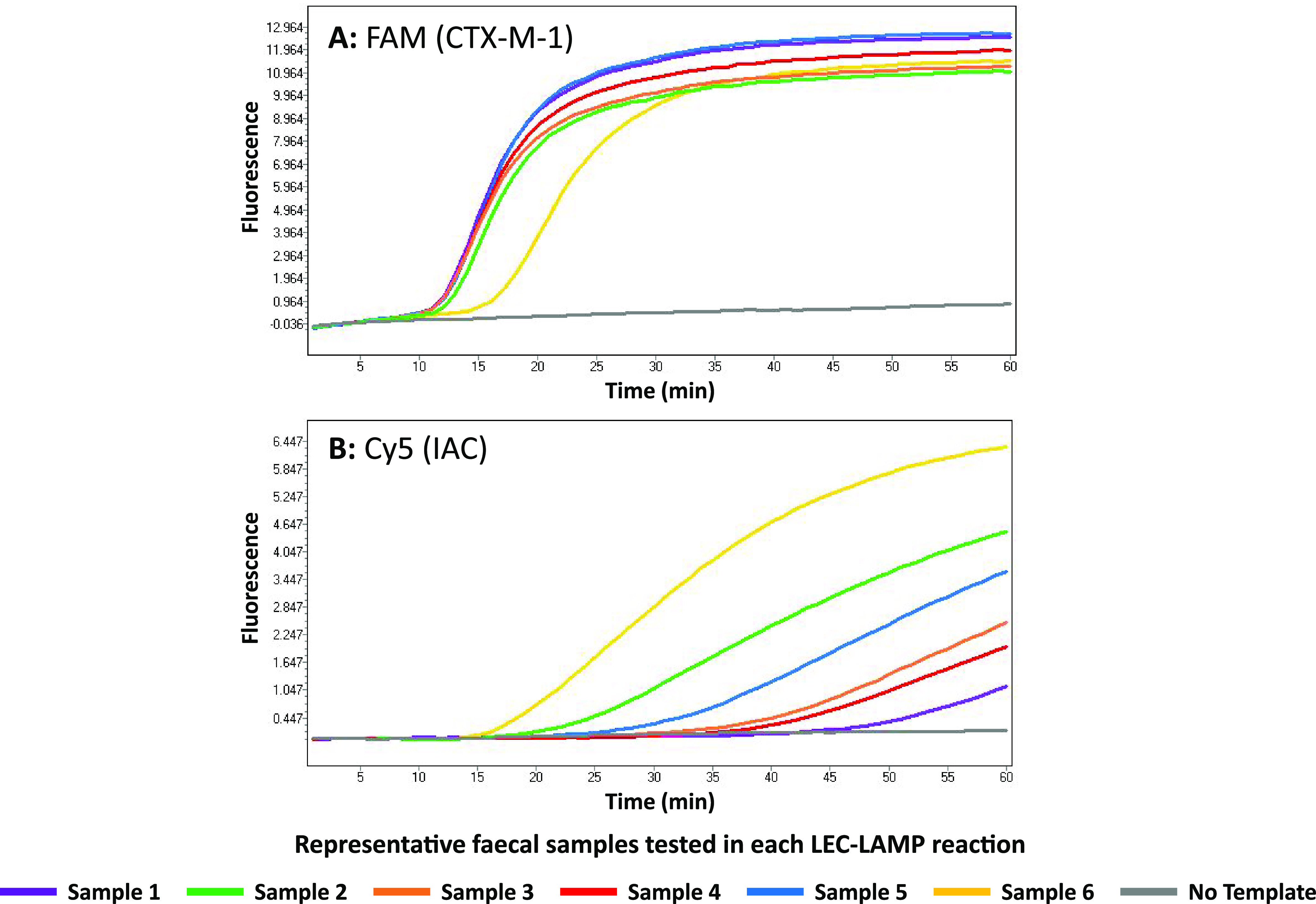
Animal fecal sample testing. The internally controlled CTX-M-1/15 LEC-LAMP assay was challenged with 2 μL DNA extracts from porcine fecal samples PCR-positive for E. coli *bla*_CTX-M_, in the presence of 500 copies IAC DNA template. Reactions were performed at 63°C in a LightCycler 480 using fluorescence detection channels: FAM, CTX-M-1 specific (A); HEX, CTX-M-15 specific (not shown); and Cy5, IAC specific (B). Resulting amplification curves for a representative selection of samples tested (Samples No. 1 to 6) are shown, indicating successful *bla*_CTX-M-1_ detection in each sample. Successful IAC co-amplification and detection in each reaction was also observed. No detection was observed in the HEX (CTX-M-15 specific) detection channel and no signal was observed in the negative-control reactions.

A total of 15 porcine fecal samples PCR-positive for E. coli *bla*_CTX-M_ were processed using the rapid DNA extraction protocol followed by CTX-M-1/15 LEC-LAMP assay analysis (Table S6). The DNA extraction processing time for a single fecal sample was approximately 12 to 15 min. Analysis of 2 μL DNA extracts from each fecal sample with the internally controlled CTX-M-1/15 LEC-LAMP assay indicated successful identification of E. coli *bla*_CTX-M-1_ in all samples. Resulting LEC-LAMP amplification curves are shown for a representative selection of fecal samples (Samples No.1 to 6, Table S6) in Fig. 5. LEC-LAMP assay time to detection for each sample was under 20 min (Fig. 5A). The IAC DNA template was successfully co-amplified and detected in each sample reaction (Fig. 5B), with no signal observed in the no template control reaction (Fig. 5A and B).

### On-site animal fecal sample testing.

Successful proof-of-concept for the on-site testing of animal fecal samples using the rapid DNA extraction protocol and CTX-M-1/15 LEC-LAMP assay was demonstrated with a portable diagnostics workstation (Fig. 2 and 3). Using the mobile workstation, Chelex-100 based heat lysis DNA extractions were performed on the porcine fecal samples positive for E. coli *bla*_CTX-M-1_ (*n* = 15) followed by LEC-LAMP analysis using the ESEQuant TS4 thermostatic fluorometer. All samples analyzed on-site produced similar positive results to the previous PCR and LEC-LAMP laboratory analysis (Table S6), resulting in the detection of *bla*_CTX-M-1_ in each sample, with successful simultaneous IAC and NTC reactions. The on-site processing time for each fecal sample from DNA extraction to LEC-LAMP result was approximately 45 min to 1 h.

## DISCUSSION

CTX-M enzymes produced by ESBL-*Enterobacteriaceae* confer resistance to third-generation cephalosporins, antibiotics considered critically important to both human and animal health, contributing to a rapidly increasing AMR global health crisis (5, 8, 27). CTX-M group 1 variants, CTX-M-1 and CTX-M-15, are the predominant ESBL-expressing *Enterobacteriaceae* associated with animal and human infection, respectively (6). E. coli is a primary host for the CTX-M group 1 gene variants, *bla*_CTX-M-1_ and *bla*_CTX-M-15_, with major source locations for these ESBL-producing pathogens being hospitals, livestock, companion animals, and related environmental settings (13, 14). Timely and accurate diagnostics are required for effective identification and monitoring of *bla*_CTX-M-1_ and *bla*_CTX-M-15_; however, with largely similar nucleotide sequences effective differential detection using conventional methods is challenging. LEC-LAMP is a recently developed isothermal nucleic acid diagnostic method facilitating rapid real-time multiplex pathogen detection with single-base specificity (26). We have developed an internally controlled multiplex CTX-M-1/15 LEC-LAMP assay for the specific identification of *bla*_CTX-M-1_ and *bla*_CTX-M-15_, evaluating this assay in terms of analytical specificity and sensitivity, and application for on-site fecal sample analysis.

Real-time detection and differentiation of *bla*_CTX-M-1_ and *bla*_CTX-M-15_ gene variants was exemplified in this study using the internally controlled CTX-M-1/15 LEC-LAMP assay (Fig. 4). In these reactions, specific differential detection of E. coli *bla*_CTX-M-1_ and E. coli *bla*_CTX-M-15_ was observed, with each variant only identified in respective fluorescence channels and low-level detection achieved in approximately 20 min. Additionally, IAC templates were successfully co-amplified and detected in the relevant specific corresponding channel. It can be seen in these reactions that the increasing concentrations of E. coli *bla*_CTX-M-1_ and E. coli *bla*_CTX-M-15_ from 10^1^, 10^2^, and 10^3^ copies caused expected minor co-amplification inhibition of the 500 copies IAC DNA template. Reactions containing the lowest bacterial template concentrations produced the earliest IAC fluorescent signals, and conversely, reactions with the highest bacterial template concentrations produced the latest IAC fluorescent signals. IAC templates incorporated into nucleic acid diagnostics only require detection in the event of no target identification (28); however, it can be observed in Fig. 4 that the IAC was confidently detected in each reaction demonstrating efficient assay robustness. This successful demonstration of robust, fast, low-level specific differential detection of *bla*_CTX-M-1_ and *bla*_CTX-M-15_ highlights a significant improvement on existing diagnostic technologies used for identification of these CTX-M group 1 variants. Typically, CTX-M-1 and CTX-M-15 phenotyping and genotyping is performed by antibiotic susceptibility testing followed by CTX-M group specific PCR and DNA sequencing (29–32), a considerably lengthier and more expensive process than the LEC-LAMP assay approach. Various studies have reported the use of PCR followed by melt analysis for CTX-M-1 and CTX-M-15 differentiation (33–35), however, these methods require postamplification analysis producing ambiguous results that require discrimination of near identical melt curves often only differing by less than 0.5°C. Further highlighting the utility of this LEC-LAMP assay, Leflon-Guibout and colleagues previously reported a CTX-M-15 specific PCR assay that cannot effectively differentiate CTX-M-15 from CTX-M-1 based on available related nucleotide sequence information (36). Johnson et al. later modified this previous assay by adding the required forward primer 5′-end guanine residue to enable CTX-M-15 variant specific PCR amplification (37).

Analytical specificity testing of the CTX-M-1/15 LEC-LAMP assay using human, animal, and environmental E. coli isolates highlighted specific differential identification of *bla*_CTX-M-1_ and *bla*_CTX-M-15_ (Table S3). However, nonspecific detection of one exclusivity panel isolate was observed with CTX-M-55 detected in the HEX (CTX-M-15 specific) channel. This result was expected as the nucleotide sequence for CTX-M-55 is identical to CTX-M-15 apart from minor isolated 3′-end SNPs. CTX-M-55 is the second most common ESBL subtype in *Enterobacteriaceae* of human and animal origin in various Asian regions, exceeding that of CTX-M-15 in some parts of China (38). First reported in E. coli and Klebsiella pneumoniae in Thailand in 2006 (39), CTX-M-55 is a member of CTX-M group 1 and a hypothesized derivative of CTX-M-15 (40) due to an identical gene sequence apart from nucleotide 239 resulting in the single amino acid substitution of valine in place of alanine at position 80 (41). The Ala-80-Val substitution, previously referred to as Ala-77-Val, is associated with higher catalytic activity of cephalosporins compared with CTX-M-15, and thus, reduced susceptibility to antibiotics such as ceftazidime (40). However, occurrence of CTX-M-55-producing *Enterobacteriaceae* is primarily restricted to Asian countries, with only sporadic reports in other regions (42, 43). Also, previous studies have demonstrated that both CTX-M-15 and CTX-M-55 ESBL-expressing E. coli isolates show susceptibility to fluoroquinolone antibiotics, ciprofloxacin and enrofloxacin (41, 44), indicating that differential detection of these variants is not essential in terms of treatment. Further to this point, beta-lactamase inhibitors and aminoglycosides have been reported as viable treatment options for both CTX-M-15 and CTX-M-55 related infections (45–47). However, for epidemiological monitoring purposes, the CTX-M-1/15 LEC-LAMP assay was designed to facilitate incorporation of an additional LEC-LAMP probe enabling potential differential detection of CTX-M-55 from CTX-M-1 and CTX-M-15. The Ala-80-Val associated nucleotide is located on the forward loop region of the CTX-M-1/15 LEC-LAMP assay (Table S1), enabling conversion of the current LF primer to a CTX-M-55 specific LEC-LAMP probe using an alternative fluorophore to the LB–CTX-M-1 and LB–CTX-M-15 probes.

Analytical sensitivity analysis of the internally controlled CTX-M-1/15 LEC-LAMP assay was performed by testing E. coli *bla*_CTX-M-1_ and E. coli *bla*_CTX-M-15_ purified DNA at 32, 16, 8, 4, 2, and 1 copies per reaction, analyzing six replicates of each concentration (Table S4). Resulting data used in combination with probit analysis indicated an LOD with 95% confidence of 8.5 and 9.8 copies per reaction for *bla*_CTX-M-1_ and *bla*_CTX-M-15_, respectively. A minimum number of six replicates per concentration is required to produce a statistically valid LOD, the lowest target concentration at which 95% of positive samples are detected (48, 49). Previous singleplex CTX-M-1 LAMP assays have reported LODs of 0.1 pg genomic DNA (50, 51), equivalent to 18 *bla*_CTX-M-1_ copies per reaction, highlighting the superior LOD of the multiplex internally controlled CTX-M-1/15 LEC-LAMP assay.

Animal porcine fecal samples were processed using a rapid DNA extraction protocol incorporating heat lysis with Chelex-100 and Tris-EDTA treatment. Chelex-100 is a styrene-divinylbenzene co-polymer that acts as a chelating material which coordinates to DNase cofactor magnesium cations, protecting extracted DNA from degradation (52). The 10% Chelex-100 sodium form solution used was preheated to aid fecal sample dispersion, and Tris-EDTA enabled necessary sample dilution and neutralization to prevent LAMP reaction inhibition. DNA extracts from each processed porcine fecal sample were confirmed positive for E. coli *bla*_CTX-M_ using a CTX-M group 1 PCR assay and these samples were further confirmed positive for E. coli *bla*_CTX-M-1_ using the CTX-M-1/15 LEC-LAMP assay (Table S5 and S6). Compared with the PCR assay, the LEC-LAMP assay demonstrated 100% diagnostic sensitivity for each fecal sample tested. Resulting PCR Ct values for each fecal sample ranged from 22 to 33, indicating the possibility of variable bacterial loads which is reflected in the representative LEC-LAMP assay results shown in Fig. 5. Sample 1, producing the lowest PCR Ct value of 22.55 indicates a high bacterial load demonstrates the earliest LEC-LAMP detection time, while sample 6, producing the highest PCR Ct value of 33.80 indicates a lower bacterial load demonstrates the latest LEC-LAMP detection time. Conversely, due to co-amplification inhibition as previously discussed in Fig. 4, IAC LEC-LAMP results for these respective samples indicated earlier IAC detection from samples with lower bacterial loads, and later IAC detection from samples with higher bacterial loads. However, although IAC template detection is not required in the event of positive target detection (28), the IAC was successfully codetected with each fecal sample analyzed, further indicating robust assay performance.

On-site analysis of the E. coli *bla*_CTX-M-1_ positive porcine fecal samples, using the custom portable diagnostics workstation (Fig. 2 and 3) incorporating the rapid DNA extraction method and internally controlled CTX-M-1/15 LEC-LAMP assay, reconfirmed the previous PCR and LEC-LAMP analysis results for each sample (Table S6). This result highlighted a successful proof-of-concept demonstration for the portable on-site application of the mobile diagnostics workstation. The portability of this novel diagnostic technology is an essential attribute for epidemiological monitoring due the varied source locations of these CTX-M variants.

The internally controlled multiplex CTX-M-1/15 LEC-LAMP assay detailed in this study is the first report of portable nucleic acid diagnostics technology applied to the differential detection of AMR markers, *bla*_CTX-M-1_ and *bla*_CTX-M-15_. This novel transferable diagnostic technology can be applied to other relevant AMR gene markers of interest providing more efficient and specific portable detection over current diagnostic methodologies for improved epidemiological surveillance. Advantages of this system over conventional AMR molecular diagnostic methods include portability, rapid time-to-detection (10 to 20 min), single-base specificity, and provision of multiplex internally controlled reactions contained within a closed-tube system. Additionally, the portability and low power requirements of this diagnostic technology are suited to application in low-resourced areas where AMR burden is typically most prevalent (53). The main limitation of this current CTX-M-1/15 LEC-LAMP assay is the requirement for reaction cold storage during transportation for on-site testing; however, the application of freeze-drying assay lyophilization would enable long-term ambient temperature reaction storage.

## MATERIALS AND METHODS

### Diagnostic targets and LEC-LAMP oligonucleotides.

The target nucleotide sequences used for CTX-M-1/15 LEC-LAMP assay design were generated from a range of environmental E. coli isolates positive for *bla*_CTX-M-1_ or *bla*_CTX-M-15_. A total of 26 E. coli environmental isolates previously collected as part of routine seawater and wastewater analysis at the University of Galway were processed using a combination of culture isolation followed by Illumina short-read sequencing to identify each isolate as positive for *bla*_CTX-M-1_ (*n* = 2) or *bla*_CTX-M-15_ (*n* = 24). Resulting E. coli *bla*_CTX-M-1_ and E. coli *bla*_CTX-M-15_ nucleotide sequences for each isolate were aligned using Clustal Omega (Table S1) and used for LEC-LAMP assay design. The target nucleotide sequence used for design of the internal amplification control (IAC) LEC-LAMP assay was a randomly generated synthetic 500 bp DNA gBlock Gene Fragment (Table S2) purchased from Integrated DNA Technologies (Munich, Germany). LEC-LAMP assay design was performed using a combination of PrimerExplorer V5 and manual design (Table 1). Unmodified oligonucleotides were synthesized using standard desalting by Integrated DNA Technologies (Germany) and modified oligonucleotides were synthesized using HPLC purification by Metabion International AG (Planegg, Germany). The modified oligonucleotide probe sequences, LB–CTX-M-1, LB–CTX-M-15, and LB–IAC, contain 5′-end BHQ1 or BHQ2 quencher labels, a 1’ 2’-dideoxyribose spacer modification in place of an internal nucleotide, and an internal thymine residue labeled FAM, HEX or Cy5 fluorophore. The fluorophores used correspond to one of three fluorescence detection channels in the LightCycler 480 Instrument II (Roche Diagnostics, Sussex, UK) and ESEQuant TS4 thermostatic fluorometer (Qiagen, Stockach, Germany) used to perform all LEC-LAMP reactions. The LB–CTX-M-1 and LB–CTX-M-15 probes were designed around the *bla*_CTX-M-1_ and *bla*_CTX-M-15_ mismatches at nucleotides 313 and 315 (Table S1). Nucleotide differences between LB–CTX-M-1 and LB–CTX-M-15 are highlighted in Table 1, and nucleotide mismatches between LB–CTX-M-1 and *bla*_CTX-M-15_, and LB–CTX-M-15 and *bla*_CTX-M-1_, are highlighted in Fig. 1.

### Bacterial DNA preparation.

All E. coli isolates used for assay development and evaluation (Table S3) were stored at −80°C on glycerol bead stocks or in extracted DNA form. Culturing of animal and environmental E. coli isolates was performed using brain heart infusion media (Oxoid, Hampshire, UK) with incubation at 37°C for 18 h, aerobically. Bacterial DNA extraction and quantification was carried out using the DNeasy blood and tissue kit (Qiagen, Hilden, Germany) and Qubit dsDNA broad range kit (Life Technologies, Warrington, UK), as per manufacturer’s instructions. All human E. coli isolates were supplied in extracted DNA form from the Royal College of Surgeons, Ireland, and the National Reference Laboratory of Antibiotic Resistances and Healthcare Associated Infections, National Institute of Health Dr. Ricardo Jorge, Portugal. These isolates were selected from human strain collections recovered from urine, blood, pus, and biopsy specimens of different hospitals. All patient samples were anonymized and not analyzed for human DNA; thus, ethical approval was not required. Gene target copy number values for each isolate were determined using resulting DNA concentrations with the assumption that 0.5 ng E. coli *bla*_CTX-M-1_ or E. coli *bla*_CTX-M-15_ purified genomic DNA extract corresponds to 90,000 copies with one copy per bacterium (54, 55).

### CTX-M-1/15 LEC-LAMP assay.

The CTX-M-1/15 LEC-LAMP assay contained 1× Isothermal Amplification Buffer (New England Biolabs, Hitchin, UK), 6 mM MgSO_4_ (Roche Diagnostics), 1.8 mM deoxynucleotide triphosphate set (New England Biolabs), CTX-M-1/15 LEC-LAMP oligonucleotides (1.6 μM FIP, 1.6 μM BIP, 0.2 μM F3, 0.2 μM B3, 0.4 μM LF, 0.4 μM LB–CTX-M-1, 0.4 μM LB–CTX-M-15), IAC LEC-LAMP oligonucleotides (1.6 μM FIP, 1.6 μM BIP, 0.2 μM F3, 0.2 μM B3, 0.4 μM LF, 0.4 μM LB–IAC), 8 U *Bst* 2.0 WarmStart DNA polymerase (New England Biolabs), 1 U Endonuclease IV (New England Biolabs), 500 copies IAC DNA gBlock Gene Fragment template, 2 μL bacterial DNA template for test reactions or 2 μL molecular grade water for no template control (NTC) reactions, and molecular grade water to give a final reaction volume of 25 μL. LEC-LAMP reactions were performed in duplicate at 63°C for 1 h in a LightCycler 480 instrument II (Roche Diagnostics). Fluorescence measurements were recorded every min using the FAM (495 to 520 nm), HEX (535 to 565 nm) and Cy5 (646 to 662 nm) detection channels producing amplification curves representing positive reactions as signal acquisition exceeding background fluorescence.

### CTX-M-1/15 LEC-LAMP assay target detection and differentiation.

Demonstration of the real-time specific differential detection and identification of CTX-M group 1 variants, CTX-M-1 and CTX-M-15, was performed using purified DNA from E. coli isolates positive for *bla*_CTX-M-1_ or *bla*_CTX-M-15_. Reducing concentrations of E. coli *bla*_CTX-M-1_ and E. coli *bla*_CTX-M-15_ genomic DNA was used to challenge the internally controlled CTX-M-1/15 LEC-LAMP assay. In separate reactions, E. coli *bla*_CTX-M-1_ and E. coli *bla*_CTX-M-15_ DNA was tested at 10^3^, 10^2^, and 10^1^ copies per reaction in the presence of 500 copies IAC DNA template. A no template control reaction using molecular grade water in place of bacterial DNA template was performed in parallel.

### CTX-M-1/15 LEC-LAMP assay analytical specificity and sensitivity.

Analytical specificity of the internally controlled CTX-M-1/15 LEC-LAMP assay was established using a panel of human, animal, and environmental E. coli isolates (Table S3). The specificity panel of isolates tested comprised of an inclusivity panel containing E. coli isolates positive for *bla*_CTX-M-1_ (*n* = 18) and *bla*_CTX-M-15_ (*n* = 35), with an exclusivity panel of E. coli isolates positive for other closely related *bla*_CTX-M_ variants (*n* = 38). Purified genomic DNA from each E. coli isolate was tested with the CTX-M-1/15 LEC-LAMP assay at concentrations of 10^4^ genome copies per reaction with no template control reactions carried out in parallel.

Analytical sensitivity of the internally controlled CTX-M-1/15 LEC-LAMP assay was determined by testing replicates of serially diluted E. coli *bla*_CTX-M-1_ and E. coli *bla*_CTX-M-15_ purified genomic DNA in the presence of 500 copies IAC DNA template. In separate reactions, E. coli *bla*_CTX-M-1_ and E. coli *bla*_CTX-M-15_ DNA was tested at 32, 16, 8, 4, 2, and 1 copies per reaction in replicates of six (Table S4). Resulting data were used to perform probit regression analysis and establish the assay limit of detection (LOD) with 95% confidence.

### Laboratory animal fecal sample testing.

Application of the internally controlled CTX-M-1/15 LEC-LAMP assay for animal fecal sample testing was demonstrated using porcine fecal samples and a rapid 7DNA extraction protocol. Porcine fecal samples were supplied by the Friedrich-Loeffler-Institut, Germany, and the University of Tartu, Estonia. Each fecal sample was processed using a rapid DNA extraction protocol involving the transfer of approximately 200 mg fecal material to a 1.5-mL tube containing 1 mL preheated (99°C) 10% Chelex-100 sodium form solution (Sigma-Aldrich, Dublin, Ireland). Fecal sample suspensions were vortexed for 30 s, incubated at 99°C for 10 min, vortexed for 30 s, and 5 μL sample lysates were transferred to 45 μL 1× Tris-EDTA (10 mM Tris-HCl, 1 mM EDTA, pH 8) (Sigma). To confirm the presence of E. coli *bla*_CTX-M_ in each fecal sample, 2 μL DNA extracts were tested using a CTX-M group 1 PCR assay (Table S5). Fecal samples confirmed PCR-positive for E. coli *bla*_CTX-M_ (*n* = 15) were further tested with the CTX-M-1/15 LEC-LAMP assay.

### On-site animal fecal sample testing.

Proof-of-concept for on-site animal fecal sample testing using the internally controlled CTX-M-1/15 LEC-LAMP assay in combination with the rapid DNA extraction protocol was performed with a custom portable diagnostics workstation (Fig. 2). The previous PCR-confirmed E. coli *bla*_CTX-M_ positive porcine fecal samples (*n* = 15) were analyzed on-site in a nonlaboratory agricultural setting using the portable diagnostics workstation which included an ESEQuant TS4 thermostatic fluorometer (Qiagen), an SLS Flowgen mini dry bath (Analab, Antrim, UK), an SLS mini vortex (Analab), a Yeti 500× Portable Power Station (Goal Zero, UT, USA), and a protector case (Peli, Clare, Ireland). Accessory items included LevGo smartSpatula (Thermo Fisher Scientific, Cork, Ireland), 1.5-mL tubes with 1 mL 10% Chelex-100 sodium form solution (Sigma), 1.5-mL tubes with 45 μL 1× Tris-EDTA (Sigma), manual single-channel 20-μL pipette with tips, and CTX-M-1/15 LEC-LAMP assay reaction mix in 0.2-mL flat cap reaction tubes (Thermo Fisher Scientific) stored on ice during transportation. On-site DNA extractions were carried out as previously outlined (Fig. 3), with LEC-LAMP reactions performed on the ESEQuant TS4 thermostatic fluorometer. Time required to transport the mobile diagnostics workstation and samples to the on-site testing location was approximately 45 min.
