# Supplementary material for: Portable Differential Detection of CTX-M ESBL Gene Variants, blaCTX-M-1 and blaCTX-M-15, from Escherichia coli Isolates and Animal Fecal Samples Using Loop-Primer Endonuclease Cleavage Loop-Mediated Isothermal Amplification
Source: Microbiol Spectr. 2022 Dec 13;11(1):e03316-22. doi: 10.1128/spectrum.03316-22 (PMC9927312; doi:10.1128/spectrum.03316-22)
Supplement: Supplemental file 1 — Supplemental material. Download spectrum.03316-22-s0001.pdf, PDF file, 0.4 MB [file spectrum.03316-22-s0001.pdf]

Supplemental Data

Table S1: Alignment of *bla*$_{CTX\text{-}M\text{-}1}$ and *bla*$_{CTX\text{-}M\text{-}15}$ nucleotide sequences isolated from *E. coli* environmental samples

```
                           10        20        30        40        50        60        70        80        90       100
                   ....|....|....|....|....|....|....|....|....|....|....|....|....|....|....|....|....|....|....|....|
1.   12394         ATGGTTAAAAAATCACTGCGTCAGTTCACGCTGATGGCGACGGCAACCGTCACGCTGTTGTTAGGAAGTGTGCCGCTGTATGCGCAAACGGCGGACGTAC
2.   6012400       ....................................................................................................
3.   6012390       ....................C...............................................................................
4.   6012392       ....................C...............................................................................
5.   6012395       ....................C...............................................................................
6.   6012396       ....................C...............................................................................
7.   6012397       ....................C...............................................................................
8.   6012399       ....................C...............................................................................
9.   6012402       ....................C...............................................................................
10.  6012403       ....................C...............................................................................
11.  6012405       ....................C...............................................................................
12.  6012406       ....................C...............................................................................
13.  6012407       ....................C...............................................................................
14.  6012382/6694  ....................C...............................................................................
15.  6012383       ....................C...............................................................................
16.  6012387/6697  ....................C...............................................................................
17.  6012366/6693  ....................C...............................................................................
18.  6012368       ....................C...............................................................................
19.  6012369       ....................C...............................................................................
20.  6012371       ....................C...............................................................................
21.  6012372       ....................C...............................................................................
22.  6012373       ....................C...............................................................................
23.  6012374       ....................C...............................................................................
24.  6012377       ....................C...............................................................................
25.  6012380       ....................C...............................................................................
26.  6012381       ....................C...............................................................................
                   ****************************************************************************************************

                          110       120       130       140       150       160       170       180       190       200
                   ....|....|....|....|....|....|....|....|....|....|....|....|....|....|....|....|....|....|....|....|
1.   12394         AGCAAAAACTTGCCGAATTAGAGCGGCAGTCGGGAGGAAGACTGGGTGTGGCATTGATTAACACAGCAGATAATTCGCAAATACTTTATCGTGCTGATGA
2.   6012400       ....................................................................................................
3.   6012390       .....................................C..............................................................
4.   6012392       .....................................C..............................................................
5.   6012395       .....................................C..............................................................
6.   6012396       .....................................C..............................................................
7.   6012397       .....................................C..............................................................
8.   6012399       .....................................C..............................................................
9.   6012402       .....................................C..............................................................
10.  6012403       .....................................C..............................................................
11.  6012405       .....................................C..............................................................
12.  6012406       .....................................C..............................................................
13.  6012407       .....................................C..............................................................
14.  6012382/6694  .....................................C..............................................................
15.  6012383       .....................................C..............................................................
16.  6012387/6697  .....................................C..............................................................
17.  6012366/6693  .....................................C..............................................................
18.  6012368       .....................................C..............................................................
19.  6012369       .....................................C..............................................................
20.  6012371       .....................................C..............................................................
21.  6012372       .....................................C..............................................................
22.  6012373       .....................................C..............................................................
23.  6012374       .....................................C..............................................................
24.  6012377       .....................................C..............................................................
25.  6012380       .....................................C..............................................................
26.  6012381       .....................................C..............................................................
                   ****************************************************************************************************

                          210       220       230       240       250       260       270       280       290       300
                   ....|....|....|....|....|....|....|....|....|....|....|....|....|....|....|....|....|....|....|....|
1.   12394         GCGCTTTGCGATGTGCAGCACCAGTAAAGTGATGGCCGTGGCCGCGGTGCTGAAGAAAAGTGAAAGCGAACCGAATCTGTTAAATCAGCGAGTTGAGATC
2.   6012400       ....................................................................................................
3.   6012390       ......................................C.............................................................
4.   6012392       ......................................C.............................................................
5.   6012395       ......................................C.............................................................
6.   6012396       ......................................C.............................................................
7.   6012397       ......................................C.............................................................
8.   6012399       ......................................C.............................................................
9.   6012402       ......................................C.............................................................
10.  6012403       ......................................C.............................................................
11.  6012405       ......................................C.............................................................
12.  6012406       ......................................C.............................................................
13.  6012407       ......................................C.............................................................
14.  6012382/6694  ......................................C.............................................................
15.  6012383       ......................................C.............................................................
16.  6012387/6697  ......................................C.............................................................
17.  6012366/6693  ......................................C.............................................................
18.  6012368       ......................................C.............................................................
19.  6012369       ......................................C.............................................................
20.  6012371       ......................................C.............................................................
21.  6012372       ......................................C.............................................................
22.  6012373       ......................................C.............................................................
23.  6012374       ......................................C.............................................................
24.  6012377       ......................................C.............................................................
25.  6012380       ......................................C.............................................................
26.  6012381       ......................................C.............................................................
                   ****************************************************************************************************

                          310       320       330       340       350       360       370       380       390       400
                   ....|....|....|....|....|....|....|....|....|....|....|....|....|....|....|....|....|....|....|....|
1.   12394         AAAAAATCTGACTTGGTTAACTATAATCCGATTGCGGAAAAGCACGTCGATGGGACGATGTCACTGGCTGAGCTTAGCGCGGCCGCGCTACAGTACAGCG
2.   6012400       ....................................................................................................
3.   6012390       ............C.T.................................A...................................................
4.   6012392       ............C.T.................................A...................................................
5.   6012395       ............C.T.................................A...................................................
6.   6012396       ............C.T.................................A...................................................
7.   6012397       ............C.T.................................A...................................................
8.   6012399       ............C.T.................................A...................................................
9.   6012402       ............C.T.................................A...................................................
10.  6012403       ............C.T.................................A...................................................
11.  6012405       ............C.T.................................A...................................................
12.  6012406       ............C.T.................................A...................................................
13.  6012407       ............C.T.................................A...................................................
14.  6012382/6694  ............C.T.................................A...................................................
15.  6012383       ............C.T.................................A...................................................
16.  6012387/6697  ............C.T.................................A...................................................
17.  6012366/6693  ............C.T.................................A...................................................
18.  6012368       ............C.T.................................A...................................................
19.  6012369       ............C.T.................................A...................................................
```

```
20.   6012371        ............C.T.................................A...................................................
21.   6012372        ............C.T.................................A...................................................
22.   6012373        ............C.T.................................A...................................................
23.   6012374        ............C.T.................................A...................................................
24.   6012377        ............C.T.................................A...................................................
25.   6012380        ............C.T.................................A...................................................
26.   6012381        ............C.T.................................A...................................................
                     ****************************************************************************************************

                            410       420       430       440       450       460       470       480       490       500
                     ....|....|....|....|....|....|....|....|....|....|....|....|....|....|....|....|....|....|....|....|
1.    12394          ATAACGTGGCGATGAATAAGCTGATTTCTCACGTTGGCGGCCCGGCTAGCGTCACCGCGTTCGCCCGACAGCTGGGAGACGAAACGTTCCGTCTCGACCG
2.    6012400        ....................................................................................................
3.    6012390        ..........................G.........................................................................
4.    6012392        ..........................G.........................................................................
5.    6012395        ..........................G.........................................................................
6.    6012396        ..........................G.........................................................................
7.    6012397        ..........................G.........................................................................
8.    6012399        ..........................G.........................................................................
9.    6012402        ..........................G.........................................................................
10.   6012403        ..........................G.........................................................................
11.   6012405        ..........................G.........................................................................
12.   6012406        ..........................G.........................................................................
13.   6012407        ..........................G.........................................................................
14.   6012382/6694   ..........................G.........................................................................
15.   6012383        ..........................G.........................................................................
16.   6012387/6697   ..........................G.........................................................................
17.   6012366/6693   ..........................G.........................................................................
18.   6012368        ..........................G.........................................................................
19.   6012369        ..........................G.........................................................................
20.   6012371        ..........................G.........................................................................
21.   6012372        ..........................G.........................................................................
22.   6012373        ..........................G.........................................................................
23.   6012374        ..........................G.........................................................................
24.   6012377        ..........................G.........................................................................
25.   6012380        ..........................G.........................................................................
26.   6012381        ..........................G.........................................................................
                     ****************************************************************************************************

                            510       520       530       540       550       560       570       580       590       600
                     ....|....|....|....|....|....|....|....|....|....|....|....|....|....|....|....|....|....|....|....|
1.    12394          TACCGAGCCGACGTTAAACACCGCCATTCCGGGCGATCCGCGTGATACCACTTCACCTCGGGCAATGGCGCAAACTCTGCGTAATCTGACGCTGGGTAAA
2.    6012400        ....................................................................................................
3.    6012390        .................................................................................G..................
4.    6012392        .................................................................................G..................
5.    6012395        .................................................................................G..................
6.    6012396        .................................................................................G..................
7.    6012397        .................................................................................G..................
8.    6012399        .................................................................................G..................
9.    6012402        .................................................................................G..................
10.   6012403        .................................................................................G..................
11.   6012405        .................................................................................G..................
12.   6012406        .................................................................................G..................
13.   6012407        .................................................................................G..................
14.   6012382/6694   .................................................................................G..................
15.   6012383        .................................................................................G..................
16.   6012387/6697   .................................................................................G..................
17.   6012366/6693   .................................................................................G..................
18.   6012368        .................................................................................G..................
19.   6012369        .................................................................................G..................
20.   6012371        .................................................................................G..................
21.   6012372        .................................................................................G..................
22.   6012373        .................................................................................G..................
23.   6012374        .................................................................................G..................
24.   6012377        .................................................................................G..................
25.   6012380        .................................................................................G..................
26.   6012381        .................................................................................G..................
                     ****************************************************************************************************

                            610       620       630       640       650       660       670       680       690       700
                     ....|....|....|....|....|....|....|....|....|....|....|....|....|....|....|....|....|....|....|....|
1.    12394          GCATTGGGTGACAGCCAACGGGCGCAGCTGGTGACATGGATGAAAGGCAATACCACCGGTGCAGCGAGCATTCAGGCTGGACTGCCTGCTTCCTGGGTTG
2.    6012400        ....................................................................................................
3.    6012390        ........C...........................................................................................
4.    6012392        ........C...........................................................................................
5.    6012395        ........C...........................................................................................
6.    6012396        ........C...........................................................................................
7.    6012397        ........C...........................................................................................
8.    6012399        ........C...........................................................................................
9.    6012402        ........C...........................................................................................
10.   6012403        ........C...........................................................................................
11.   6012405        ........C...........................................................................................
12.   6012406        ........C...........................................................................................
13.   6012407        ........C...........................................................................................
14.   6012382/6694   ........C...........................................................................................
15.   6012383        ........C...........................................................................................
16.   6012387/6697   ........C...........................................................................................
17.   6012366/6693   ........C...........................................................................................
18.   6012368        ........C...........................................................................................
19.   6012369        ........C...........................................................................................
20.   6012371        ........C...........................................................................................
21.   6012372        ........C...........................................................................................
22.   6012373        ........C...........................................................................................
23.   6012374        ........C...........................................................................................
24.   6012377        ........C...........................................................................................
25.   6012380        ........C...........................................................................................
26.   6012381        ........C...........................................................................................
                     ****************************************************************************************************

                            710       720       730       740       750       760       770       780       790       800
                     ....|....|....|....|....|....|....|....|....|....|....|....|....|....|....|....|....|....|....|....|
1.    12394          TGGGGGATAAAACCGGCAGCGGTGACTATGGCACCACCAACGATATCGCGGTGATCTGGCCAAAAGATCGTGCGCCGCTGATTCTGGTCACTTACTTCAC
2.    6012400        ....................................................................................................
3.    6012390        ........................G...........................................................................
4.    6012392        ........................G...........................................................................
5.    6012395        ........................G...........................................................................
6.    6012396        ........................G...........................................................................
7.    6012397        ........................G...........................................................................
8.    6012399        ........................G...........................................................................
9.    6012402        ........................G...........................................................................
10.   6012403        ........................G...........................................................................
11.   6012405        ........................G...........................................................................
12.   6012406        ........................G...........................................................................
13.   6012407        ........................G...........................................................................
14.   6012382/6694   ........................G...........................................................................
15.   6012383        ........................G...........................................................................
16.   6012387/6697   ........................G...........................................................................
17.   6012366/6693   ........................G...........................................................................
18.   6012368        ........................G...........................................................................
19.   6012369        ........................G...........................................................................
20.   6012371        ........................G...........................................................................
21.   6012372        ........................G...........................................................................
22.   6012373        ........................G...........................................................................
```

```
23.  6012374         ........................G...........................................................................
24.  6012377         ........................G...........................................................................
25.  6012380         ........................G...........................................................................
26.  6012381         ........................G...........................................................................
                     ****************************************************************************************************

                             810       820       830       840       850       860       870
                     ....|....|....|....|....|....|....|....|....|....|....|....|....|....|....|.
1.   12394           CCAGCCTCAACCTAAGGCAGAAAGCCGTCGCGATGTATTAGCGTCGGCGGCTAAAATCGTCACCAACGGTTTGTAA
2.   6012400         ............................................................----------------
3.   6012390         ................................................................G...........
4.   6012392         ................................................................G...........
5.   6012395         ................................................................G...........
6.   6012396         ................................................................G...........
7.   6012397         ................................................................G...........
8.   6012399         ................................................................G...........
9.   6012402         ................................................................G...........
10.  6012403         ................................................................G...........
11.  6012405         ................................................................G...........
12.  6012406         ................................................................G...........
13.  6012407         ................................................................G...........
14.  6012382/6694    ................................................................G...........
15.  6012383         ................................................................G...........
16.  6012387/6697    ................................................................G...........
17.  6012366/6693    ................................................................G...........
18.  6012368         ................................................................G...........
19.  6012369         ................................................................G...........
20.  6012371         ................................................................G...........
21.  6012372         ................................................................G...........
22.  6012373         ................................................................G...........
23.  6012374         ................................................................G...........
24.  6012377         ................................................................G...........
25.  6012380         ................................................................G...........
26.  6012381         ................................................................G...........
                     **************************************************
```

1-2, *E. coli bla*$_{CTX-M-1}$ nucleotide sequences; 3-26, *E. coli bla*$_{CTX-M-15}$ nucleotide sequences; red, CTX-M-1/15 LEC-LAMP assay outer and inner primer locations; underline, CTX-M-1/15 LEC-LAMP assay loop primer locations.

Table S2: Internal amplification control (IAC) synthetic DNA template nucleotide sequence (5' to 3')

```
CGGCGCAGTGCTGCCGACAGCCGGGCATTGTCTTTGGGGCGTTATTCGAGGGCACCCGGACCTAACTTGTCGGGACCACCCGGGGTAGTCATCG
GGCTTATACAGCGAAAAGCCCAGCACCCGGCTCCCCGCTATGGAAGGTCATTAGCTCCGGCAAGCAATTAAGAACAACGCAAGGATCGCGGATA
TAAACAGAGAAACGGCCGAATACACCTGTTCGTGTCGTATCGGTAAATAGACTCTCATGCGCAATGATGGTATAATCCTGCGGAGCACTCTGGT
AATGCATATGGTCCACAGGACATTCGTCGCTTCCGGGTATGCGCTCTATGTGACGGTCTTTTGGCGCACAAATGCTCAGCACCATTTAAATTAG
ACCGACTCCAGATCTGTAAGGTCCGCCACGCAGACGACAGCCCACGGAGACCACTGACCGATCTACCTGAACGGCGACCATCTGTGTGGTACTG
GGGCGGAGAGATAACTACGGTGCCGCTTAC
```

Table S3: CTX-M-1/15 LEC-LAMP assay specificity panel

Organism	Strain Identifier	Source	Supplier	LEC-LAMP Result		
				CTX-M-1 (FAM)	CTX-M-15 (HEX)	IAC (Cy5)
**Inclusivity Panel**						
CTX-M-1 isolates						
E. coli	Isolate #27	Municipal wastewater	NUIG	+	-	+
E. coli	Isolate #80	Hospital sewage	NUIG	+	-	+
E. coli	BH229	River water	NUIG	+	-	+
E. coli	B19229	River water	NUIG	+	-	+
E. coli	B19444	Seawater	NUIG	+	-	+
E. coli	B19476	Seawater	NUIG	+	-	+
E. coli	B19684	Municipal wastewater	NUIG	+	-	+
E. coli	B19678	Seawater	NUIG	+	-	+
E. coli	EC17	Human	RCSI	+	-	+
E. coli	EC22	Human	RCSI	+	-	+
E. coli	EC29	Human	RCSI	+	-	+
E. coli	EC53	Human	RCSI	+	-	+
E. coli	20E0033	Porcine	FLI	+	-	+
E. coli	20E0041	Porcine	FLI	+	-	+
E. coli	20E0104	Porcine	FLI	+	-	+
E. coli	20E0490	Alpaca	FLI	+	-	+
E. coli	20E0508	Alpaca	FLI	+	-	+
E. coli	20E0539	Alpaca	FLI	+	-	+
CTX-M-15 isolates						
E. coli	BH160	Seawater	NUIG	-	+	+
E. coli	BH170	Seawater	NUIG	-	+	+
E. coli	BH224	Seawater	NUIG	-	+	+
E. coli	Isolate #10	Hospital sewage	NUIG	-	+	+

E. coli	Isolate #15	Hospital sewage	NUIG	-	+	+
E. coli	Isolate #32	Hospital sewage	NUIG	-	+	+
E. coli	Isolate #48	Hospital sewage	NUIG	-	+	+
E. coli	Isolate #60	Municipal wastewater	NUIG	-	+	+
E. coli	Isolate #63	Municipal wastewater	NUIG	-	+	+
E. coli	Isolate #97	Seawater	NUIG	-	+	+
E. coli	Isolate #106	Municipal wastewater	NUIG	-	+	+
E. coli	Isolate #130	Municipal wastewater	NUIG	-	+	+
E. coli	Isolate #117	Hospital sewage	NUIG	-	+	+
E. coli	Isolate #139	Municipal wastewater	NUIG	-	+	+
E. coli	Isolate #97	Seawater	NUIG	-	+	+
E. coli	Strain A	Seawater	NUIG	-	+	+
E. coli	RF2	Veterinary	UCD	-	+	+
E. coli	RF6	Veterinary	UCD	-	+	+
E. coli	RF9	Veterinary	UCD	-	+	+
E. coli	RF11	Veterinary	UCD	-	+	+
E. coli	RF12	Veterinary	UCD	-	+	+
E. coli	RF14	Veterinary	UCD	-	+	+
E. coli	RF16	Veterinary	UCD	-	+	+
E. coli	9487	Veterinary	UCD	-	+	+
E. coli	4212	Veterinary	UCD	-	+	+
E. coli	EC36	Human	RCSI	-	+	+
E. coli	EC37	Human	RCSI	-	+	+
E. coli	EC51	Human	RCSI	-	+	+
E. coli	EC62	Human	RCSI	-	+	+
E. coli	EC76	Human	RCSI	-	+	+
E. coli	EC83	Human	RCSI	-	+	+
E. coli	EC85	Human	RCSI	-	+	+
E. coli	EC86	Human	RCSI	-	+	+
E. coli	EC90	Human	RCSI	-	+	+
E. coli	20E0012	Porcine	FLI	-	+	+
**Exclusivity Panel**						
Other closely related CTX-M isolates						
E. coli	CTX-M-55	Porcine	FLI	-	+	+
E. coli	CTX-M-27	Porcine	FLI	-	-	+
E. coli	CTX-M	Seawater	NUIG	-	-	+
E. coli	CTX-M	Seawater	NUIG	-	-	+
E. coli	CTX-M	Seawater	NUIG	-	-	+
E. coli	IMP-type	Reference strain	NUIG	-	-	+
E. coli	CTX-M-2	Reference strain	NUIG	-	-	+
E. coli	CTX-M-8	Reference strain	NUIG	-	-	+
E. coli	CTX-M-9	Reference strain	NUIG	-	-	+
E. coli	CTX-M-25	Reference strain	NUIG	-	-	+
E. coli	CTX-M-26	Reference strain	NUIG	-	-	+
E. coli	7604 CTX-M-9	Human	INSA	-	-	+
E. coli	14674 CTX-M-9	Human	INSA	-	-	+
E. coli	16856 CTX-M-9	Human	INSA	-	-	+
E. coli	17104-A CTX-M-9	Human	INSA	-	-	+
E. coli	17104-B CTX-M-9	Human	INSA	-	-	+
E. coli	17117 CTX-M-9	Human	INSA	-	-	+
E. coli	17121 CTX-M-9	Human	INSA	-	-	+
E. coli	6119 CTX-M-14	Human	INSA	-	-	+
E. coli	6122 CTX-M-14	Human	INSA	-	-	+
E. coli	6132 CTX-M-14	Human	INSA	-	-	+
E. coli	6189 CTX-M-14	Human	INSA	-	-	+
E. coli	6190 CTX-M-14	Human	INSA	-	-	+
E. coli	6322 CTX-M-14	Human	INSA	-	-	+
E. coli	7593 CTX-M-14	Human	INSA	-	-	+
E. coli	7607 CTX-M-14	Human	INSA	-	-	+
E. coli	7663 CTX-M-14	Human	INSA	-	-	+

E. coli	20590 CTX-M-14	Human	INSA	-	-	+
E. coli	20994 CTX-M-14	Human	INSA	-	-	+
E. coli	21005 CTX-M-14	Human	INSA	-	-	+
E. coli	21024 CTX-M-14	Human	INSA	-	-	+
E. coli	21049 CTX-M-14	Human	INSA	-	-	+
E. coli	20950 CTX-M-27	Human	INSA	-	-	+
E. coli	20954 CTX-M-27	Human	INSA	-	-	+
E. coli	20957 CTX-M-27	Human	INSA	-	-	+
E. coli	20996 CTX-M-27	Human	INSA	-	-	+
E. coli	21015 CTX-M-27	Human	INSA	-	-	+
E. coli	21050 CTX-M-27	Human	INSA	-	-	+

NUIG, National University of Ireland, Galway; RCSI, Royal College of Surgeons, Ireland; FLI, Friedrich-Loeffler-Institut, Germany; UCD, University College Dublin; INSA, Instituto Nacional de Saúde Dr. Ricardo Jorge, Portugal; +, positive; -, negative.

Table S4: CTX-M-1/15 LEC-LAMP assay limit of detection (LOD) probit analysis

Copy number concentration	CTX-M-1	CTX-M-15
	Tested / Detected	Tested / Detected
32	6 / 6	6 / 6
16	6 / 6	6 / 6
8	6 / 6	6 / 5
4	6 / 2	6 / 4
2	6 / 1	6 / 1
1	6 / 0	6 / 0
**LOD**	**8.5**	**9.8**

Table S5: CTX-M group 1 PCR assay*

**Primer**	**Sequence (5’ to 3’)**
Forward	GACAGCTGGGAGACGAAAC
Reverse	ATTGCCCGAGGTGAAGTG

* Reactions contained 1X LightCycler 480 SYBR Green 1 Master, 0.5 µM forward primer, 0.5 µM reverse primer, 1 µL DNA template or molecular grade water for no template control reactions, and molecular grade water to give a final reaction volume of 20 µL. Reactions were performed using a LightCycler 480 and cycling parameters of 95°C for 5 min followed by 45 cycles of 60°C for 15 s, 72°C for 15 s and 95°C for 10 s, with fluorescence acquisition recorded in the FAM detection channel during each elongation step.

Table S6: Porcine faecal sample testing using CTX-M group 1 PCR assay and CTX-M-1/15 LEC-LAMP assay

Sample No.	Sample Identifier	Sample Supplier	CTX-M group 1 PCR Result (Ct Value)	LEC-LAMP Result					
				LightCycler 480			ESEQuant TS4		
				CTX-M-1 (FAM)	CTX-M-15 (HEX)	IAC (Cy5)	CTX-M-1 (FAM)	CTX-M-15 (HEX)	IAC (Cy5)
1	19SC0430	FLI	+ (22.55)	+	-	+	+	-	+
2	20SC0063	FLI	+ (26.53)	+	-	+	+	-	+
3	20SC0007	FLI	+ (27.76)	+	-	+	+	-	+
4	20SC0008	FLI	+ (26.71)	+	-	+	+	-	+
5	20SC0058	FLI	+ (29.46)	+	-	+	+	-	+
6	20SC0006	FLI	+ (33.80)	+	-	+	+	-	+
7	Vo-1	UT	+ (28.56)	+	-	+	+	-	+
8	Vo-2	UT	+ (30.04)	+	-	+	+	-	+
9	Vo-3	UT	+ (28.82)	+	-	+	+	-	+

10	Vo-4	UT	+ (29.79)	+	-	+	+	-	+
11	Ra-1	UT	+ (26.70)	+	-	+	+	-	+
12	Ra-2	UT	+ (27.19)	+	-	+	+	-	+
13	Ra-3	UT	+ (26.84)	+	-	+	+	-	+
14	Ra-4	UT	+ (26.77)	+	-	+	+	-	+
15	Mä-1	UT	+ (29.64)	+	-	+	+	-	+

FLI, Friedrich-Loeffler-Institut, Germany; UT, University of Tartu, Estonia; +, positive; -, negative.
